# ^225^Ac-MACROPATATE: A Novel α-Particle Peptide Receptor Radionuclide Therapy for Neuroendocrine Tumors

**DOI:** 10.2967/jnumed.122.264707

**Published:** 2023-04

**Authors:** A. Paden King, Nicholas T. Gutsche, Natarajan Raju, Stanley Fayn, Kwamena E. Baidoo, Meghan M. Bell, Colleen S. Olkowski, Rolf E. Swenson, Frank I. Lin, Samira M. Sadowski, Stephen S. Adler, Nikki A. Thiele, Justin J. Wilson, Peter L. Choyke, Freddy E. Escorcia

**Affiliations:** 1 Molecular Imaging Branch, Center for Cancer Research, National Cancer Institute, National Institutes of Health, Bethesda, Maryland;; 2 Chemical and Synthesis Center, National Heart, Lung, and Blood Institute, National Institutes of Health, Bethesda, Maryland;; 3 Surgical Oncology Program, Center for Cancer Research, National Cancer Institute, National Institutes of Health, Bethesda, Maryland;; 4 Clinical Research Directorate, Frederick National Laboratory for Cancer Research, Frederick, Maryland;; 5 Chemical Sciences Division, Oak Ridge National Laboratory, Oak Ridge, Tennessee;; 6 Department of Chemistry and Chemical Biology, Baker Laboratory, Cornell University, Ithaca, New York; and; 7 Radiation Oncology Branch, Center for Cancer Research, National Cancer Institute, National Institutes of Health, Bethesda, Maryland

**Keywords:** oncology, actinium, targeted α-therapy, neuroendocrine tumors, octreotate, somatostatin

## Abstract

Neuroendocrine tumors (NETs) express somatostatin receptors (SSTRs) 2 and 5. Modified variants of somatostatin, the cognate ligand for SSTR2 and SSTR5, are used in treatment for metastatic and locoregional disease. Peptide receptor radionuclide therapy with ^177^Lu-DOTATATE (DOTA-octreotate), a β-particle–emitting somatostatin derivative, has demonstrated survival benefit in patients with SSTR-positive NETs. Despite excellent results, a subset of patients has tumors that are resistant to treatment, and alternative agents are needed. Targeted α-particle therapy has been shown to kill tumors that are resistant to targeted β-particle therapy, suggesting that targeted α-particle therapy may offer a promising treatment option for patients with ^177^Lu-DOTATATE–resistant disease. Although DOTATATE can chelate the clinically relevant α-particle–emitting radionuclide ^225^Ac, the labeling reaction requires high temperatures, and the resulting radioconjugate has suboptimal stability. **Methods:** We designed and synthesized MACROPATATE (MACROPA-octreotate), a novel radioconjugate capable of chelating ^225^Ac at room temperature, and assessed its in vitro and in vivo performance. **Results:** MACROPATATE demonstrated comparable affinity to DOTATATE (dissociation constant, 21 nM) in U2-OS-SSTR2, a SSTR2-positive transfected cell line. ^225^Ac-MACROPATATE demonstrated superior serum stability at 37°C over time compared with ^225^Ac-DOTATATE. Biodistribution studies demonstrated higher tumor uptake of ^225^Ac-MACROPATATE than of ^225^Ac-DOTATATE in mice engrafted with subcutaneous H69 NETs. Therapy studies showed that ^225^Ac-MACROPATATE exhibits significant antitumor and survival benefit compared with saline control in mice engrafted with SSTR-positive tumors. However, the increased accumulation of ^225^Ac-MACROPATATE in liver and kidneys and subsequent toxicity to these organs decreased its therapeutic index compared with ^225^Ac-DOTATATE. **Conclusion:** ^225^Ac-MACROPATATE and ^225^Ac-DOTATATE exhibit favorable therapeutic efficacy in animal models. Because of elevated liver and kidney accumulation and lower administered activity for dose-limiting toxicity of ^225^Ac-MACROPATATE, ^225^Ac-DOTATATE was deemed the superior agent for targeted α-particle peptide receptor radionuclide therapy.

## Visual Abstract

**Figure:**
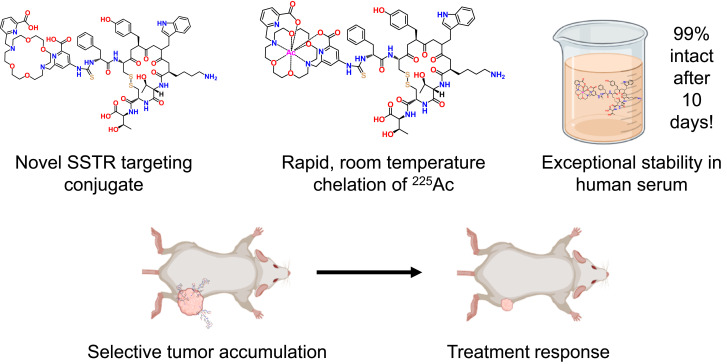

Neuroendocrine tumors (NETs) are a heterogeneous family of neoplasms originating in cells within the endocrine and nervous systems that reside in the gastrointestinal tract, lungs, pancreas, thyroid, and gonads (1*,*^2^). Many NETs overexpress somatostatin receptors (SSTRs) (3). This high receptor expression offers a targetable vulnerability in NETs, which has long been exploited for therapy.

Somatostatin-like derivatives have been used as drugs themselves or as scaffolds to deliver radioisotopes for peptide receptor radionuclide therapy (PRRT). One of the most successful of these is the pairing of Tyr^3^-octreotate with the chelator DOTA, yielding DOTATATE (4*,*^5^). Radiolabeled DOTATATE has been successfully used for both PET imaging (6) (^68^Ga, ^64^Cu) and therapeutic (^177^Lu) purposes. The phase 3 randomized controlled clinical trial NETTER-1 showed that patients with treatment-refractory NETs who received ^177^Lu-DOTATATE had significantly better progression-free survival than patients receiving somatostatin analogs (7). The results of this trial led to FDA approval of ^177^Lu-DOTATATE (Lutathera; Advanced Accelerator Applications) in January 2018 for the treatment of SSTR-positive gastroenteropancreatic NETs (8).

Although these results made PRRT a first-in-class treatment option for patients with NETs, many are *ab initio* resistant to, or develop resistance after treatment with, β-particle–emitting ^177^Lu-DOTATATE. α-particle–emitting radionuclides are an attractive alternative to β-particle–emitting radionuclides because of their short range, which can mitigate off-target effects, and the high energy deposited by these particles over that short range (also known as high linear energy transfer, or LET) (9*,*^10^). The α-particle–emitting nuclide ^225^Ac has been coupled to prostate-specific membrane antigen (PSMA)–targeting ligands to successfully treat prostate cancers refractory to treatments with androgen deprivation, taxanes, and ^177^Lu-PSMA-617 (Pluvicto; Advanced Accelerator Applications), which is approved for treatment of patients with PSMA-positive metastatic castration-resistant prostate cancer in the United States (11–14). Recently, a phase I clinical trial of patients with gastroenteropancreatic NETs previously treated with ^177^Lu-DOTATATE and receiving ^225^Ac-DOTATATE therapy showed stable disease or a partial response in 82% of patients (15). Similarly, another study with ^225^Ac-DOTATATE found it to have efficacy in patients with SSTR-positive paraganglioma (16). α-emitting PRRT with ^213^Bi (half-life, 45 min) and ^212^Pb (half-life, 10.6 h) have shown promising clinical results as well (17*,*^18^).

DOTA and its derivatives are used to chelate ^225^Ac and many of the previously mentioned radionuclides. However, to chelate ^225^Ac with DOTA to yield high-specific-activity radioconjugates, temperatures above 70°C are typically required. Even if these temperatures are used, the resulting complex’s thermodynamic stability (19) and labeling kinetics are suboptimal (20). Thiele *et al*. showed that MACROPA, an 18-membered macrocycle, is capable of chelating ^225^Ac at room temperature more quickly and at lower concentrations than DOTA (21). The ^225^Ac-MACROPA complex showed comparable stability (8 d) to ^225^Ac-DOTA in human serum and in C57BL6 mice. Further preclinical studies have demonstrated the suitability of ^225^Ac-labeled MACROPA-containing radioconjugates for targeted α-therapy with both small-molecule and antibody conjugates (22*,*^23^).

In this work, we synthesized and characterized MACROPATATE, consisting of MACROPA coupled to Tyr^3^-octreotate, and compared its performance with that of DOTATATE with respect to ^225^Ac labeling efficiency, serum stability, target engagement, and therapeutic efficacy. We found that MACROPATATE exhibits improved stability over DOTATATE when complexed to ^225^Ac, maintains high SSTR binding affinity, demonstrates favorable *in vivo* target localization, and has significant antitumor activity.

## MATERIALS AND METHODS

### Synthesis and Radiolabeling of MACROPATATE and DOTATATE

MACROPATATE and DOTATATE were prepared by conjugating isothiocyanate-activated MACROPA and DO3A-tri-tert-butyl ester, respectively, to immobilized octreotate (21*,*^24^*,*^25^). After synthesis and deprotection, the products were characterized for purity and identity by high-performance liquid chromatography and liquid chromatography–mass spectrometry, respectively. Full synthetic details for both conjugates are reported in Supplemental Figure 1 (supplemental materials are available at http://jnm.snmjournals.org). Radiolabeling of MACROPATATE and DOTATATE with ^225^Ac was performed at room temperature or 70°C, respectively, in NH_4_OAc (pH 5.5), and the products were characterized using instant thin-layer chromatography (ITLC). Full radiolabeling and characterization details are provided in the supplemental information.

### Cell Culture and In Vitro Assays

A panel of SSTR2- and SSTR5-expressing cell lines were cultured, and their SSTR2 and SSTR5 expression levels were evaluated using flow cytometry. The highly positive U2OS-SSTR2 cell line was used to confirm the binding affinity of radiolabeled ^225^Ac-MACROPATATE in a saturation binding assay. Full cell culture details and experimental procedures for flow cytometry and saturation assays are reported in the supplemental information.

### Serum Stability Studies

^225^Ac-MACROPATATE and ^225^Ac-DOTATATE were evaluated for stability in human serum (EMD Millipore) at 37°C and pH 7.4. Radiochelate was diluted to 370 kBq in 1 mL of human serum and placed on an Eppendorf ThermoMixer set to 37°C. At fixed intervals, aliquots were removed from the reactions and analyzed by ITLC as described in the supplemental information.

### Murine Subcutaneous Xenograft Models

All procedures and animal studies followed a protocol approved by the National Institutes of Health Institutional Animal Care and Use Committee (protocol ROB104). Female athymic homozygous nude mice (NCI Athymic NCr-nu/nu strain 553; Charles River Laboratories), 8–10 wk old, were subcutaneously engrafted with 8 × 10^6^ H69 cells in 200 μL of ice-cold phosphate-buffered saline. Treatment of tumors with radiotracers for biodistribution or therapy studies was performed once palpable tumors developed, approximately 1 mo after inoculation. The biodistributions of both ^225^Ac-MACROPATATE and ^225^Ac-DOTATATE were evaluated in H69 subcutaneous tumor models, both with and without d-lysine pretreatment (26). Full experimental details for the biodistribution experiments are reported in the supplemental information.

### Dose-Finding Study for ^225^Ac-MACROPATATE

To evaluate the therapeutic potential of ^225^Ac-MACROPATATE, we performed a dose-finding study on mice. Mice (*n* ≥ 3) bearing H69 tumor xenografts were first injected with d-lysine hydrochloride (35 mg/mouse) and then treated with 148, 93.3, 46.3, or 23.1 kBq of ^225^Ac-MACROPATATE; their body weights and tumor growth were monitored over several weeks. The highest tested dose of ^225^Ac-MACROPATATE was based on a recent report of therapy using ^225^Ac-DOTATATE, which found that 148 kBq was well tolerated in mice (27).

### Head-to-Head Therapy Study with ^225^Ac-MACROPATATE or ^225^Ac-DOTATATE

For our therapy study, we wished to identify the highest administered activities that exhibited acceptable toxicity as measured by mouse weight loss and survival, and we found 46.3 kBq of ^225^Ac-MACROPATATE and 148 kBq of ^225^Ac-DOTATATE to be suitable. Animals engrafted with H69 cells were treated with either of the 2 radioconjugates or saline (*n* = 8–10, each). Local control and survival were the primary outcomes. All groups were tracked for humane endpoints including, but not limited to, tumors larger than 2,000 mm^3^ and weight loss greater than 20%.

### Statistical Analysis

Statistical analysis was performed using Prism (version 9.0; GraphPad Software). Statistical analysis of survival curves was performed using the log-rank test. Comparisons of organ uptake, tumor volume, and stability were performed using the Student *t* test.

## RESULTS

### MACROPATATE Forms Stable Complex with ^225^Ac at Room Temperature

**FIGURE 1.**
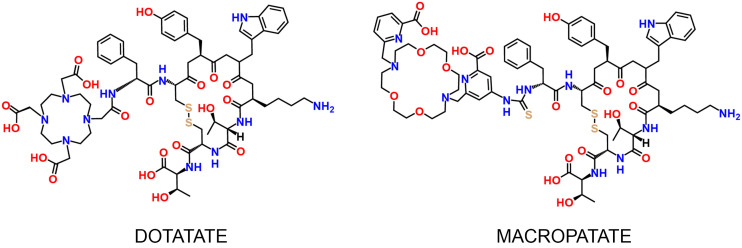
Structures of DOTATATE and MACROPATATE.

**FIGURE 2.**
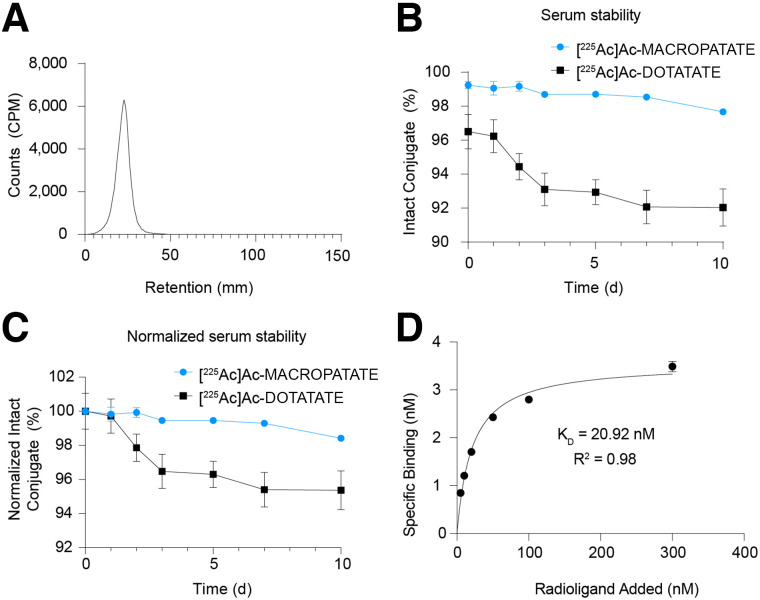
MACROPATATE stably chelates ^225^Ac and binds to SSTR. (A) Representative ITLC chromatogram of ^225^Ac-MACROPATATE. (B) Intact conjugate remaining over time of ^225^Ac-MACROPATATE and ^225^Ac-DOTATATE in human serum incubated at 37°C, as measured by ITLC. (C) Percentage of initial intact conjugate remaining over time, normalized to starting amount, after incubation in human serum at 37°C. (D) Assessment of SSTR2 binding affinity of ^225^Ac-MACROPATATE in U2-OS SSTR2 cells using saturation binding assay.

We successfully synthesized DOTATATE and MACROPATATE with good yields and high purity, characterizing the identity and purity of both molecules via high-performance liquid chromatography and mass spectrometry (Fig. 1; Supplemental Fig. 1). After synthesis, we labeled both DOTATATE and MACROPATATE with ^225^Ac. Radiolabeling was conducted in mildly acidic (pH 5.5) NH_4_OAc buffer (0.1 M). We found that MACROPATATE could be radiolabeled quantitatively after 1 h of incubation at room temperature (18–20°C), whereas DOTATATE required heating at 70°C for 1 h to achieve comparable purity and yield. Typical specific activities for both radioconjugates were approximately 185 GBq/mmol. A representative instant thin-layer chromatogram of ^225^Ac-MACROPATATE is shown in Figure 2A and Supplemental Figure 2, and an instant thin-layer chromatogram of ^225^Ac-DOTATATE is shown in Supplemental Figure 3.

We evaluated the stability of both molecules in human serum at 37°C using ITLC (Figs. 2B and 2C; Supplemental Figs. 4–7). ^225^Ac-MACROPATATE and ^225^Ac-DOTATATE were stable to 10 d. Stability experiments conducted in human serum indicated that ^225^Ac-MACROPATATE has significantly greater stability than ^225^Ac-DOTATATE (98% vs. 95%, *P* = 0.0097), and our results compared well with a recent stability investigation of ^225^Ac-DOTATATE reported by others, which found 90% intact ^225^Ac-DOTATATE after 10 d (27). Thus, ^225^Ac-MACROPATATE exhibited a modest yet significant stability advantage over ^225^Ac-DOTATATE.

### ^225^Ac-MACROPATATE Retains Affinity for SSTR

After evaluating the purity and stability of the ^225^Ac-MACROPATATE conjugate, we sought to confirm its binding affinity using SSTR2-expressing cells in vitro (28–30). Saturation binding assays showed a binding affinity of 21 nM (Fig. 2D), comparable to that reported for Eu-DOTATATE (22 nM), which has been used as a surrogate for ^225^Ac-labeled DOTATATE (27). This value is also in a similar range to other studies examining radiopeptide somatostatin derivatives (31*,*^32^). These results confirmed that MACROPA conjugation to octreotate does not adversely affect SSTR binding.

### Cell Lines Were Selected for In Vivo Studies

**FIGURE 3.**
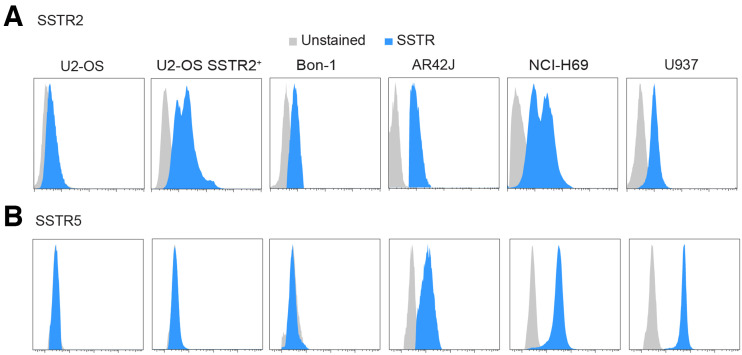
Flow cytometry assessment of SSTR2 (A) and SSTR5 (B) in panel of SSTR-expressing cell lines.

To find a suitable model for our murine subcutaneous xenograft models, we assessed SSTR2 and SSTR5 expression by flow cytometry in several cell lines (U2-OS, U2-OS-SSTR2, AR42J, H69, Bon-1, and U937) (Fig. 3). US-OS exhibited low to negligible levels of both SSTR2 and SSTR5. All other cell lines were SSTR2-positive, with expression decreasing in the order U2OS-SSTR2 > H69 > AR42J > U937 > Bon-1. The cell lines H69, AR42J, and U937 also displayed moderate expression of SSTR5. The H69 cell line was chosen for *in vivo* experiments because of its high expression of SSTR2 and SSTR5 and its established history as a model system for investigating SSTR-targeting radioconjugates (33*,*^34^).

### ^225^Ac-MACROPATATE Demonstrates Target Engagement *In Vivo*

**FIGURE 4.**
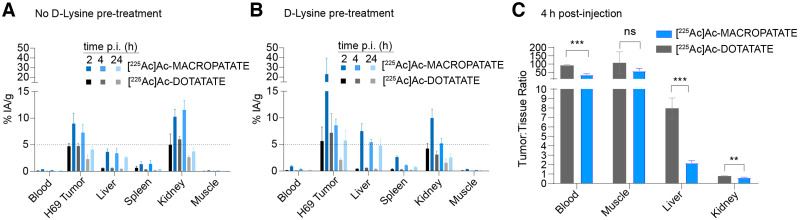
^225^Ac-labeled MACROPATATE and DOTATATE bind to SSTR-positive tumors. (A and B) Selected-organ biodistribution (*n* = 3) of ^225^Ac-MACROPATATE and ^225^Ac-DOTATATE (37 kBq) without (A) or with (B) preadministration of d-lysine (35 mg/mouse). (C) Corresponding tumor-to-tissue ratios for selected organs with lysine pretreatment. Full 12-organ biodistribution data are reported in Supplemental Figures 8–11. Error bars represent SD. ***P < 0.005. *P < 0.01. %IA/g = percentage injected activity per gram of tissue.

After confirming the stability and SSTR binding of ^225^Ac-MACROPATATE *in vitro*, we evaluated its biodistribution and compared it with that of ^225^Ac-DOTATATE in mice bearing SSTR-positive H69 tumor xenografts. Both tracers showed favorable tumor uptake, with percentage injected activity per gram of tissue of 9% and 5% at 2 h and 4% and 2% at 24 h for ^225^Ac-MACROPATATE and ^225^Ac-DOTATATE, respectively (Figs. 4A and 4B; Supplemental Figs. 8–11). Although both tracers displayed excellent tumor-to-muscle ratios of more than 50:1 at 4 h (Fig. 4C), they also had high renal uptake, as is typical of SSTR-targeting peptides (35*,*^36^). ^225^Ac-MACROPATATE displayed significantly higher tumor accumulation at 2 and 24 h than did ^225^Ac-DOTATATE (*P* < 0.05). However, the liver and kidney uptake of ^225^Ac-MACROPATATE were also higher, possibly because the higher hydrophobicity of the conjugate could slow clearance, leading to increased liver uptake. The liver accumulation of ^225^Ac-MACROPATATE was 2–3 times higher than that of ^225^Ac-DOTATATE at all time points investigated.

The high kidney accumulation of peptide radioconjugates is routinely lowered by preadministration of d-lysine (26). Accordingly, we observed a lower kidney percentage injected activity per gram of tissue for both ^225^Ac-MACROPATATE and ^225^Ac-DOTATATE after d-lysine administration than in kidneys of animals not receiving d-lysine. This lowered kidney accumulation of the radiotracers was most pronounced at 4 h after injection, with the kidney signal for ^225^Ac-MACROPATATE changing from 11.5% to 5.2% (*P* = 0.0057) and ^225^Ac-DOTATATE decreasing from 6.0% to 3.1% (*P* = 0.0039).

### ^225^Ac-MACROPATATE and ^225^Ac-DOTATATE Delay Tumor Growth and Improve Survival of Mice Bearing NET Xenografts

The promising biodistribution profile of ^225^Ac-MACROPATATE led us to investigate its therapeutic efficacy. As a preliminary investigation, we evaluated a series of treatment activities of ^225^Ac-MACROPATATE ranging from 23.1 to 148 kBq in mice bearing H69 tumor xenografts. All mice in the 148-kBq treatment group (10/10) and 1 of 3 mice in the 92.3-kBq group were euthanized within 10 d of treatment because of substantial weight loss (>20%). All other mice displayed minimal weight loss, and a clear dose-dependent reduction in tumor volume was evident (Supplemental Figs. 12–14). On the basis of these results, 46.3 kBq of ^225^Ac-MACROPATATE was selected as the appropriate dose for further investigation.

**FIGURE 5.**
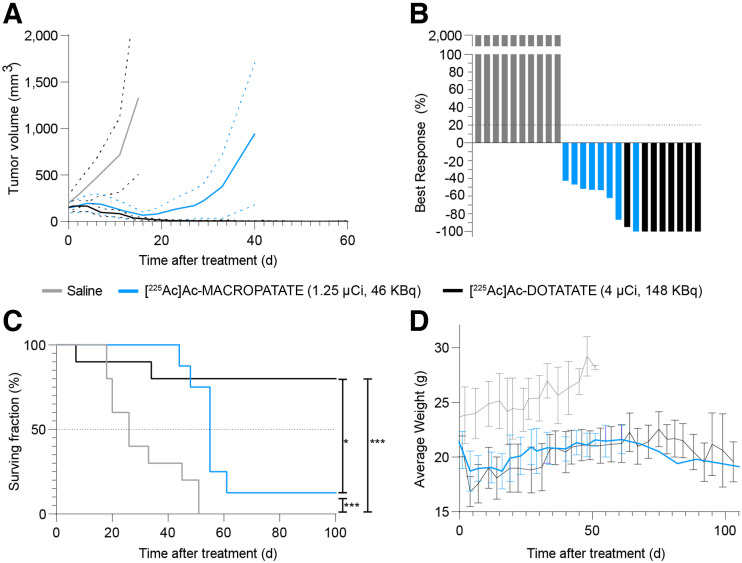
Therapeutic response of mice bearing H69 lung NET xenografts treated with ^225^Ac-MACROPATATE (46 kBq), ^225^Ac-DOTATATE (148 kBq), or vehicle control shows that targeted α-therapy with ^225^Ac-MACROPATATE and ^225^Ac-DOTATATE is effective. (A) Tumor volume measurements over time. Solid lines represent average volume, and dashed lines represent 95% CI. Plots for each dataset are discontinued after first mouse death due to excessive tumor volume. Full tumor volume measurements for study duration for each mouse are reported in Supplemental Figures 15–17. (B) Maximal response to treatment (tumor volume growth percentage) of individual mice. Nonresponding mice are represented as full tumor growth (2,000% increase). (C) Mouse survival over time. Endpoint was defined as tumor volume > 2,000 mm^3^ or weight loss over 20% of starting weight. (D) Average mouse body weights over time for each treatment group. Error bars represent SD. ***P < 0.005. *P < 0.05.

Animals treated with ^225^Ac-MACROPATATE and ^225^Ac-DOTATATE demonstrated a significant tumor growth delay and improvements in survival compared with saline-treated controls (Figs. 5A–5C; Supplemental Figs. 15–17). Mice treated with ^225^Ac-MACROPATATE exhibited an initial reduction in tumor volume lasting approximately 3 wk after treatment. However, the tumors subsequently relapsed in most mice (7 of 8). Conversely, ^225^Ac-DOTATATE treatment resulted in complete, durable tumor remission for all mice. However, 2 mice in the ^225^Ac-DOTATATE treatment group were euthanized because of weight loss. Although mice in the MACROPATATE treatment group also displayed some weight loss immediately after treatment, their weights stabilized within 2 wk (Fig. 5D). ^225^Ac-MACROPATATE significantly improved median survival relative to the vehicle control (55 d vs. 26 d; log rank, *P* = 0.0006), whereas 8 of 10 mice (80%) treated with ^225^Ac-DOTATATE survived the full 100-d duration of the study. Overall, ^225^Ac-MACROPATATE exhibited favorable local control and a survival benefit over saline-treated animals. However, mice treated with ^225^Ac-DOTATATE showed significantly better local control and overall survival (*P* < 0.02).

## DISCUSSION

PRRT with ^177^Lu-DOTATATE represents a significant advance for patients with SSTR-expressing NETs. Nevertheless, treatment options for tumors refractory to ^177^Lu-based PRRT are needed. By exploiting the unique properties of α-particles, namely high energy deposition over a short path, we may be able to overcome resistance to ^177^Lu-based PRRT (16*,*^27^*,*^37^*,*^38^). However, the DOTA chelator used in these and other studies is suboptimal for chelation of the large Ac^3+^ ion (19). With the goal of achieving a more stable SSTR-targeting radioconjugate for ^225^Ac targeted α-particle therapy, we designed, synthesized, and characterized the conjugate MACROPATATE, wherein we replace the DOTA of DOTATATE with the expanded macrocyclic chelator MACROPA, which has been shown to more stably chelate ^225^Ac^3+^ than DOTA can (21).

We confirmed that ^225^Ac-MACROPATATE displayed a high tumor accumulation, tumor growth delay, and survival benefit in xenograft models of NETs. However, our radioconjugate also exhibited a narrow therapeutic index as evinced by toxicity at lower injected activities than is the case with ^225^Ac-DOTATATE. As such, the head-to-head therapy study was performed with a 3-fold higher injected activity in animals receiving ^225^Ac-DOTATATE than in those receiving ^225^Ac-MACROPATATE. Biodistribution studies indicated a relatively high liver accumulation for ^225^Ac-MACROPATATE. Notably, unlike most other organs, this liver signal did not appear to diminish over time. This persistent accumulation of ^225^Ac may arise from metabolism of the radioconjugate and may be responsible for the observed higher toxicity of ^225^Ac-MACROPATATE. Previous investigations of ^177^Lu-DOTATATE have indicated significant degradation of the targeting octreotate portion of the tracer, likely because of metabolism (39). Such metabolism may significantly impact the biodistribution of the ^225^Ac radioconjugates explored in this work. Thus, the disparate off-target uptake of ^225^Ac-MACROPATATE and ^225^Ac-DOTATATE despite their similar tumor accumulation may also reflect accumulation of fragmented species in nontarget organs. Although ^225^Ac-MACROPATATE demonstrates significant antitumor activity in SSTR-expressing models of NET, ^225^Ac-MACROPATATE remains inferior to ^225^Ac-DOTATATE *in vivo*. Therefore, ^225^Ac-MACROPATATE requires significant optimization to decrease off-target accumulation and associated toxicity. For instance, variation of the specific activity or molar amount of ^225^Ac-MACROPATATE injected may provide decreased background accumulation while preserving tumor uptake, for such optimization has been shown to greatly improve the pharmacokinetic profile of ^177^Lu-DOTATATE (40).

Targeted α-particle therapy agents directed at NETs, such as ^225^Ac-DOTATATE, have demonstrated promising results in small clinical studies and warrant further investigation. Other α-particle–emitting radionuclides being investigated include ^213^Bi and ^212^Pb. Recently, a phase I study with the α-particle–emitting PRRT agent ^212^Pb-DOTAMTATE, which has a lead-optimized chelator, has shown good tolerability and overall response rates of 80% in patients naïve to PRRT (18). The phase II study (NCT05153772) is open and recruiting. These studies indicate that the intrinsic properties of α-emitters can elicit responses in tumors otherwise refractory to β-emitting PRRT (41*,*^42^).

More broadly, several strategies aiming to improve the efficacy of ^177^Lu-PRRT in NETs are being investigated and may apply to targeted α-particle therapy PRRT as well. For instance, deploying epigenetic modulators has been shown to increase the membrane expression of SSTR and subsequent accumulation of PRRT agent (43–46). Combinatorial approaches exploiting inhibitors of DNA damage repair are also being explored (NCT04086485, NCT04375267, and NCT03958045).

## CONCLUSION

We have successfully synthesized MACROPATATE, a novel SSTR2- and SSTR5-targeting PRRT agent tailored to deliver ^225^Ac to NETs. Importantly, we showed that MACROPATATE is able to chelate ^225^Ac at room temperature and that this complex has 4-fold lower susceptibility to degradation than ^225^Ac-DOTATATE in human serum. Both ^225^Ac-MACROPATATE and ^225^Ac-DOTATATE demonstrated excellent *in vivo* target engagement in NET xenografts and exhibited local control and survival superior to saline. However, although ^225^Ac-MACROPATATE had *in vitro* stability superior to ^225^Ac-DOTATATE, it underperformed compared with ^225^Ac-DOTATATE *in vivo*. Optimization is therefore needed before further translation.

## DISCLOSURE

This work was supported in part by the National Institutes of Biomedical Imaging and Bioengineering of the National Institutes of Health (awards R21EB027282 and R01EB029259), as well as by the National Cancer Institute from Intramural Research Program funds ZIA BC 011800 and ZIA BC 010891. This project was funded in whole or in part by the National Cancer Institute, National Institutes of Health, under contract 75N91019D00024. The content of this publication does not necessarily reflect the views or policies of the Department of Health and Human Services, nor does mention of trade names, commercial products, or organizations imply endorsement by the U.S. government. Justin Wilson and Nikki Thiele are authors of a patent for the use of MACROPA as a chelator for ^225^Ac chelation. No other potential conflict of interest relevant to this article was reported.

## Notes

KEY POINTS **QUESTION:** Can chemical conjugation of the MACROPA chelator to SSTR-targeting octreotate result in an SSTR-targeting radioconjugate for targeted α-therapy superior to DOTATATE? **PERTINENT FINDINGS:** ^225^Ac-MACROPATATE demonstrated in vitro SSTR2 affinity comparable to that of the standard ^225^Ac-DOTATATE, as well as higher in vivo uptake in SSTR-positive xenografts. However, its efficacy is limited by a poor therapeutic index, highlighting a need for further optimization before translation. **IMPLICATIONS FOR PATIENT CARE:** We confirm that targeted α-therapies for NETs demonstrate high efficacy in preclinical studies. Such agents warrant further clinical investigation to offer a therapeutic option for patients with disease refractory to β-particle PRRT.
